# Stereoselective synthesis of *trans*-fused iridoid lactones and their identification in the parasitoid wasp *Alloxysta victrix*, Part II: Iridomyrmecins

**DOI:** 10.3762/bjoc.8.141

**Published:** 2012-08-08

**Authors:** Robert Hilgraf, Nicole Zimmermann, Lutz Lehmann, Armin Tröger, Wittko Francke

**Affiliations:** 1 Department of Chemistry - Organic Chemistry, University of Hamburg, Martin-Luther-King-Platz 6, D-20146 Hamburg, Germany

**Keywords:** *Alloxysta victrix*, identification, iridoid, stereoselective synthesis, *trans*-fused iridomyrmecin

## Abstract

Following our earlier approach to the synthesis of dihydronepetalactones, all eight stereoisomers of *trans*-fused iridomyrmecins were synthesized starting from the enantiomers of limonene. Combined gas chromatography and mass spectrometry including enantioselective gas chromatography revealed that volatiles released by the endohyperparasitoid wasp *Alloxysta victrix* contain (4*S*,4a*R*,7*S*,7a*R*)-iridomyrmecin of 95–97% ee and stereochemically pure (4*S*,4a*S*,7*R*,7a*S*)-iridomyrmecin as a minor component.

## Introduction

**Figure 1:**
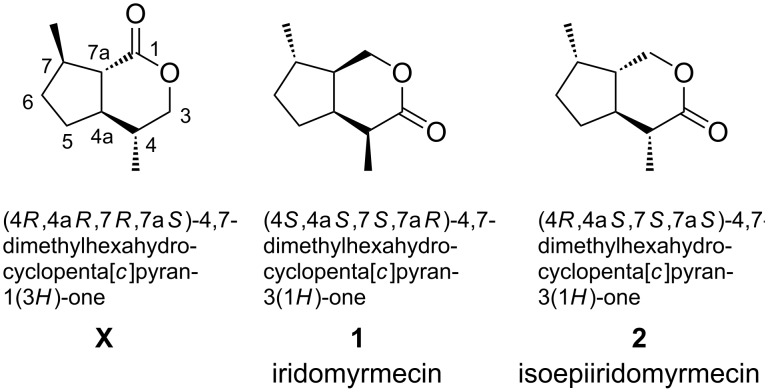
Structures of *cis*- and *trans*-fused iridoid lactones.

In the course of our studies on volatile signals of the endohyperparasitoid wasp, *Alloxysta victrix*, we identified several acyclic terpenoids and the *trans*-fused (4*S*,4a*R*,7*R*,7a*S*)-dihydronepetalactone (**X**) as volatile components of cephalic secretions released by this species (Figure 1) [1–2]. However, gas chromatograms showed the presence of two additional major volatiles **Y** and **Z** which, according to their mass spectra, were suggested to be *trans*-fused iridomyrmecins [3–4]. Since no synthetic reference compounds were available, all eight *trans*-fused iridomyrmecins had to be prepared. To complete the synthesis of this suite, we started from optically active limonene following a strategy similar to our route leading to *trans*-fused dihydronepetalactones [1]. The realization of this task and the unambiguous structure assignment of the natural products **Y** and **Z** is subject of the present paper.

## Results and Discussion

**Figure 2:**
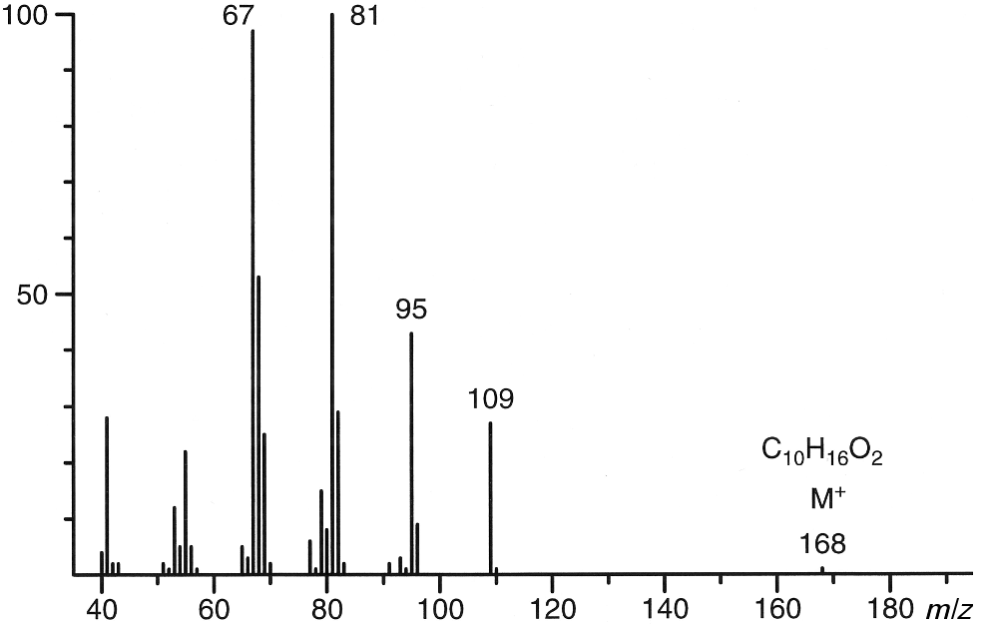
70 eV EI-mass spectrum of compounds **Y** and **Z** of *Alloxysta victrix*.

Upon coupled gas chromatography/mass spectrometry (GC/MS), two major components, **Y** and **Z**, of the volatile secretions of both sexes of *Alloxysta victrix* (although in much higher amounts in males) gave almost identical 70 eV EI-mass spectra (Figure 2); somewhat resembling that of the *trans*-fused dihydronepetalactone that we had already found in the insects [1]. Gas chromatography coupled with chemical ionization mass spectrometry (GC/CIMS) proved the molecular mass of the target compounds to be [M]^+^ = 168, while high resolution mass spectrometry (GC/HRMS) showed their atomic composition to be C_10_H_16_O_2_, confirming the compounds to be isomers of dihydronepetalactone. Though the fragmentation pattern showed some similarities to that of the *cis*-fused iridomyrmecin (**1**), a comparison with mass spectral data published for the *trans*-fused isoepiiridomyrmecin (**2**) suggested that the substances **Y** and **Z** are *trans*-fused iridomyrmecins. While the plotted mass spectrum of **1** showed *m*/*z* 95 as the base peak and similar abundances of about 55% for *m*/*z* 67, *m*/*z* 81 and *m*/*z* 109 [3], the data of **2** refer to *m*/*z* 81 as the base peak and *m*/*z* 95 and *m*/*z* 109 to reach 48% and 33%, respectively [4]; this is more close to the spectra of **Y** and **Z** (Figure 2).

Iridomyrmecin (**1**) was first isolated from ants (Figure 1) [5] and along with some other volatile iridoids it has since been reported to be a potent insecticide and antibiotic from several natural sources [6]. Due to their challenging molecular structures and their interesting physiological properties, iridomyrmecins are attractive targets in stereoselective synthesis.

**Figure 3:**
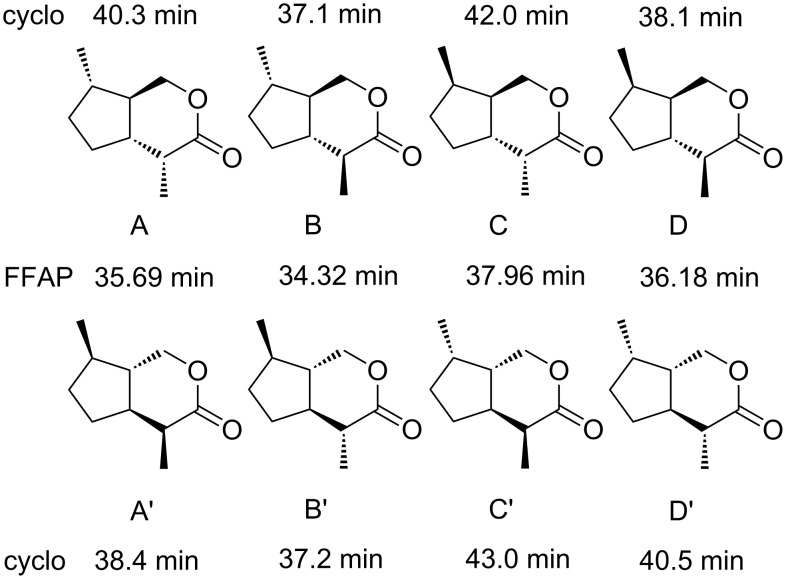
Chemical structures of all eight stereoisomers of *trans*-fused iridomyrmecins and their gas chromatographic retention times (min) on FFAP and on a cyclodextrin column (cyclo). For experimental details see Supporting Information File 1.

Similar to dihydronepetalactones, the iridomyrmecin skeleton shows four contiguous stereogenic centers giving rise to four *trans*-fused stereoisomers **A**–**D** and four corresponding enantiomers **A'**–**D'** (Figure 3, **D'** is identical to **2** in Figure 1). The presence of the four chiral centers complicates a stereoselective synthesis despite the small size of the molecule. Several methods have been published for the preparation of optically active *cis*-fused bicyclic iridoid lactones [7–10], whereas only a few syntheses of *trans*-fused ring systems have been reported [11]. Starting from enantiomerically pure (*R*)-pulegone, mixtures of (7*R*)-configured diastereomers **A'** and **B'** as well as **C** and **D** have been synthesized by Wolinsky [12]. The set of stereochemically pure (7*S*)-configured, *trans*-fused iridomyrmecins **A**, **B**, **C'**, and **D'** has been prepared by Trave [13]. Though Wolinky’s route may generally be used for the synthesis of all eight stereoisomers of *trans*-fused iridomyrmecins, it suffers from several major disadvantages such as high costs of (*S*)-pulegone and difficult separations of diastereomeric mixtures.

Starting from the cheaply available pure enantiomers of limonene, we had reported a novel stereoselective route towards *trans*-fused dihydronepatalactones [1] which we now extended to the synthesis of all eight stereoisomers of *trans*-fused iridomyrmecins. Subsequently, the volatile components **Y** and **Z** – present in *Alloxysta victrix* – were confirmed to be *trans*-fused iridomyrmecins, and their absolute configurations could be determined by comparison of their analytical data with those of all eight synthetic stereoisomers.

### Synthesis of *trans*-fused iridomyrmecins

**Figure 4:**
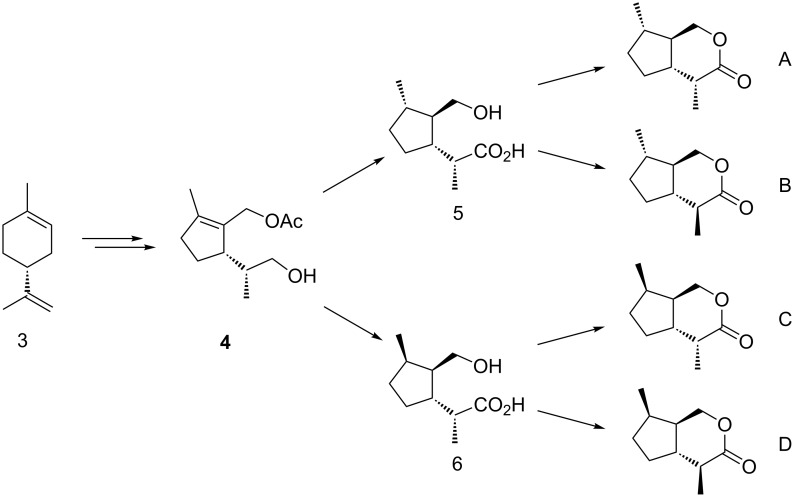
Strategy for the stereoselective synthesis of *trans*-fused iridomyrmecins **A**–**D** from (*R*)-limonene.

Our approach to the eight *trans*-fused iridomyrmecins starting from the enantiomers of limonene followed our general route for the synthesis of *trans*-fused dihydronepetalactones (Figure 4) [1].

Starting from pure (*R*)-limonene (**3**), the key intermediate **4** was prepared as described previously [1] and was used for both the synthesis of *trans,trans* and *cis,trans* (these designations refer to the relative configurations between the methyl group at C-7 and the substitution pattern at C-7a and C-4a, respectively) configured iridomyrmecins. Key steps were two stereoselective hydrogenations: A transfer hydrogenation for a formal “*anti*” delivery of hydrogen [14–15], as represented in **5**, and the use of Crabtree’s catalyst in a directed hydrogenation for a “*syn*” addition of hydrogen as represented in **6** [16–18]. Subsequent to the hydrogenation step, the synthesis of *trans*-fused iridomyrmecins could be completed after some standard functional group modifications. The synthesis of the corresponding enantiomers followed the same way, starting from (*S*)-limonene (**3'**).

### Synthesis of *trans*-fused iridomyrmecins **A** and **B**

**Scheme 1:**
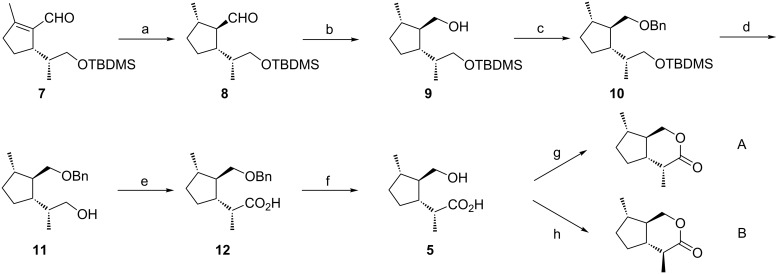
Synthesis of the *trans*-fused iridomyrmecins **A** and **B**. Reaction conditions and yields: a) ammonium formate, Pd/C, MeOH, reflux (48%); b) NaBH_4_, MeOH, H_2_O, rt (79%); c) NaH, BnBr, THF, reflux (96%); d) HF, CH_3_CN, rt (71%); e) CrO_3_, H_2_SO_4_, acetone, rt (99%); f) Pd/C, 40 bar H_2_, THF, rt (100%); g) DCC, DMAP, CH_2_Cl_2_, rt (66%); h) *p*-TsOH, benzene, reflux (56%).

The aldehyde **8**, derived from (*R*)-limonene (**3**), served as the key intermediate for the synthesis of the *trans-*fused iridomyrmecins **A** and **B** (Scheme 1). As shown in the stereoselective synthesis of *trans*-fused dihydronepetalactones [1], this key intermediate could be obtained via a highly diastereoselective transfer hydrogenation of the known [19] trisubstituted cyclopentene **7** with ammonium formate over palladium [1,14–15]. Starting from the aldehyde **8**, the relative configuration of which had been confirmed by NOE experiments [1], the synthesis of **A** and **B** was completed in six additional steps.

**Figure 5:**
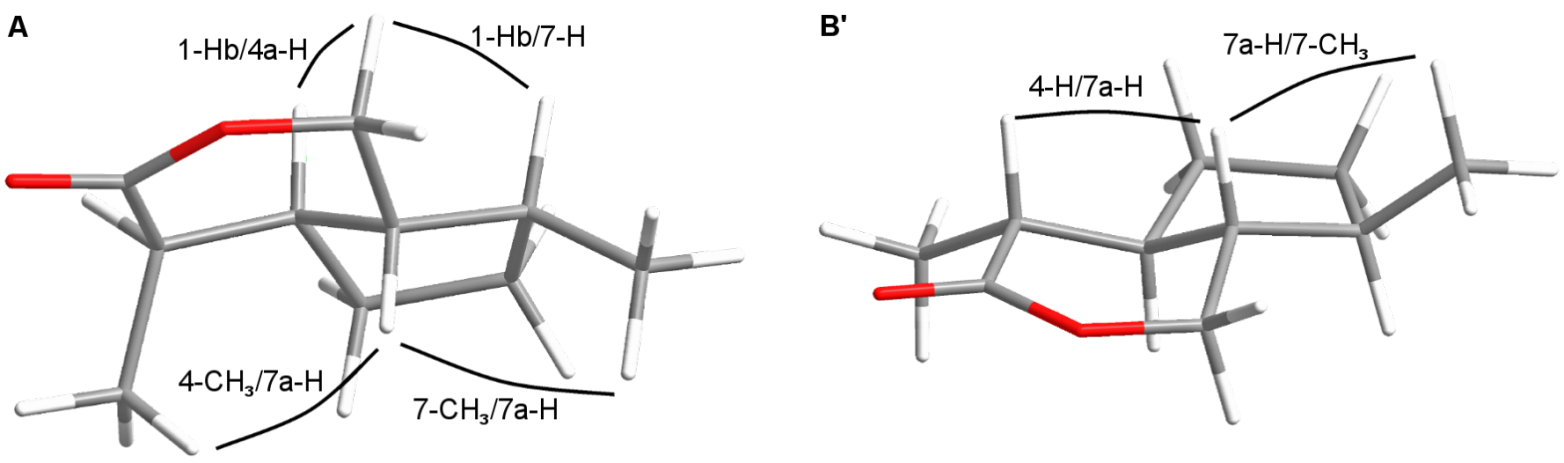
Configurations of the *trans*-fused iridomyrmecins **A** and **B’**.

First, the aldehyde **8** was reduced with sodium borohydride, and the resulting alcohol **9** was protected as the benzyl ether to form **10**. Deprotection of the TBDMS ether was carried out with HF in acetonitrile to yield the mono-protected diol **11**. Using Jones reagent, the free hydroxy group of **11** was oxidized to the carboxylic acid **12**, and the benzyl ether was cleaved upon catalytic hydrogenation over Pd/C to produce the hydroxy acid **5**. The latter served as the immediate precursor for the formation of either of the two diastereomeric iridomyrmecins **A** and **B**: Careful cyclization using dicyclohexylcarbodiimide (DCC) and 4-dimethylaminopyridine (DMAP) in dichloromethane at rt afforded iridomyrmecin **A**. In contrast, treatment of **5** with catalytic amounts of *p*-toluenesulfonic acid in benzene under reflux conditions for 12 h resulted in a complete epimerization at the CH-acidic C-4 position, exclusively yielding the thermodynamically more stable iridomyrmecin **B**. All reaction steps were also carried out starting from enantiomerically pure (*S*)-limonene affording *trans*-fused iridomyrmecins **A'** and **B'**. Relative configurations of iridomyrmecins **A**, **A'** and **B**, **B'** were confirmed by NOESY experiments. In **A** (Figure 5, A) decisive NOEs could be observed between 1-Hb and 4a-H as well as between 1-Hb and 7-H which showed 4a-H and 7-H to be located at the same side of the molecule. In addition, NOEs between 4-CH_3_ and 7a-H as well as between 7-CH_3_ and 7a-H proved the two methyl groups to be located at the same side of the molecule. In contrast, as shown in Figure 5, **B'**, NOEs between 4-H and 7a-H as well between 7-CH_3_ and 7a-H, prove that in **B'** the two methyl groups are situated at opposite sides of the molecule.

### Synthesis of *trans*-fused iridomyrmecins **C** and **D**

**Scheme 2:**
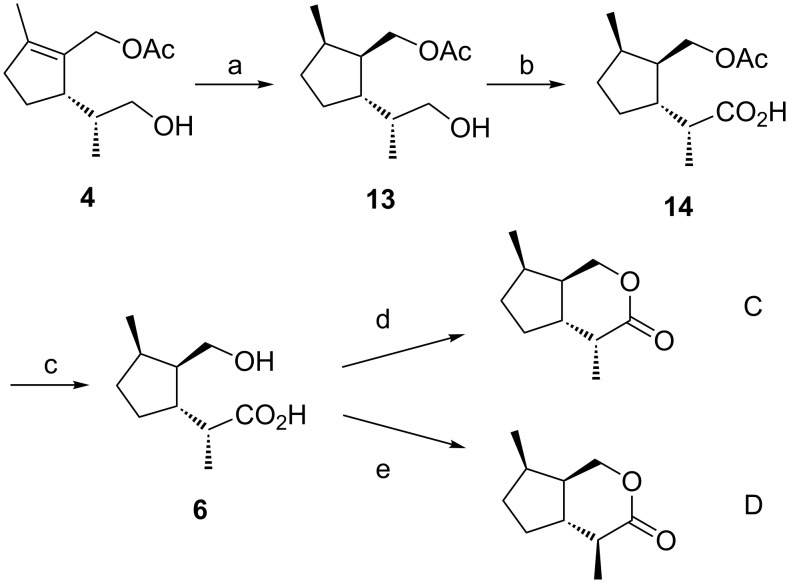
Synthesis of the *trans*-fused iridomyrmecins **C** and **D**. Reaction conditions and yields: a) Crabtree's catalyst [Ir(cod)PCy_3_(py)]PF_6_ (10 mol %), 1 bar H_2_, CH_2_Cl_2_, rt (81%); b) CrO_3_, H_2_SO_4_, acetone, rt (86%); c) KOH, MeOH, rt (97%); d) DCC, DMAP, CH_2_Cl_2_, rt (41%); e) *p*-TsOH, benzene, reflux (59%).

As shown in our previous paper on the synthesis of *trans*-fused dihydronepetalactones, the double bond of the acetate **4** could be hydrogenated with high stereocontrol to the diastereomerically pure acetate **13** [1] by using Crabtree’s catalyst [16–18]. The synthesis of the iridomyrmecins **C** and **D** was completed in three additional steps (Scheme 2). The oxidation with Jones reagent yielded **14**, and subsequent saponification of the acetate group with methanolic KOH afforded the hydroxy acid **6**. Similar to the approach described above, careful lactonization with DCC and DMAP gave iridomyrmecin **C**, whereas treatment with *p*-toluenesulfonic acid in benzene under reflux conditions led to complete epimerization at C-4 and afforded iridomyrmecin **D**. All reaction steps were also carried out starting from enantiomerically pure (*S*)-limonene (**3'**) and afforded iridomyrmecins **C'** and **D'**.

**Figure 6:**
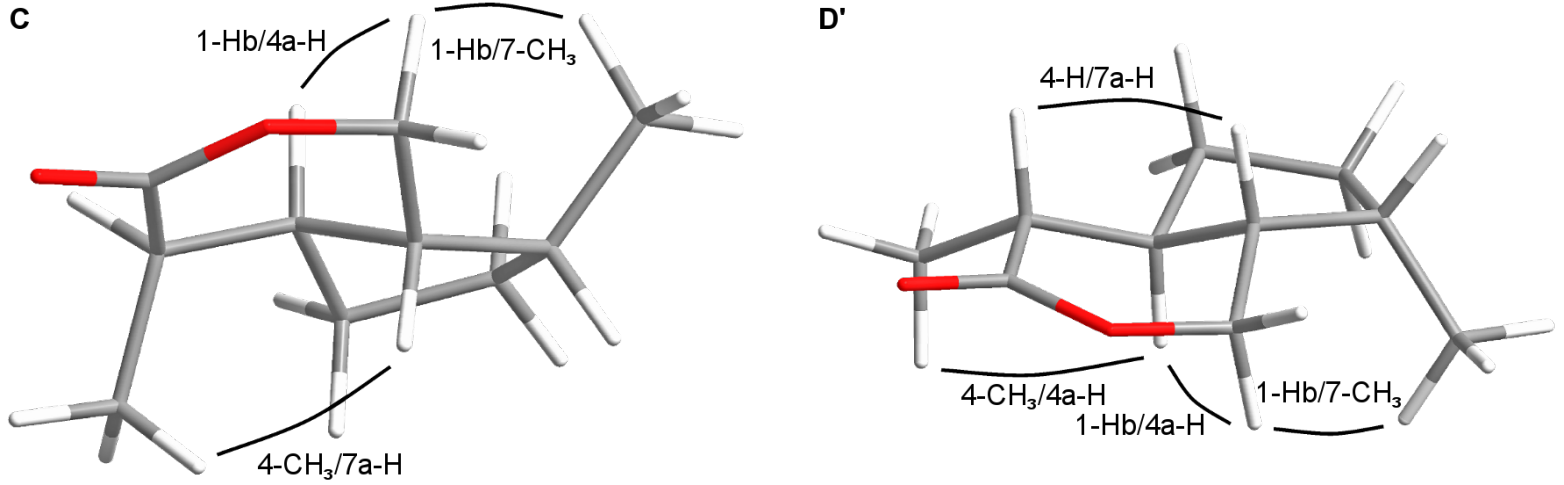
Configurations of the *trans*-fused iridomyrmecins **C** and **D’**.

Relative configurations of the iridomyrmecins **C**, **C'** and **D**, **D'** were confirmed by NOESY experiments. In **C** (Figure 6, **C**) decisive NOEs between 1-Hb and 4a-H as well as between 1-Hb and 7-CH_3_ proved 4a-H and 7-CH_3_ to be located at the same side of the molecule. The NOE between 4-CH_3_ and 7a-H showed them to be geometrically close and the two methyl groups to be in opposite positions. In contrast, as shown in Figure 6, **D'** NOEs between 4a-H and 4-CH_3_ as well as between 4a-H and 1-Hb and furthermore between 1-Hb and 7-CH_3_ proved the methyl groups in **D'** to be at the same side of the molecule. This is confirmed by a NOE between 4-H and 7a-H.

In summary, we have completed the first enantioselective synthesis of all eight stereoisomers of *trans*-fused iridomyrmecins, starting from either of the cheaply available enantiomers of limonene. The acetate **4** is the decisive intermediate, and key reaction steps are two stereoselective hydrogenations: A transfer hydrogenation for a formal “*anti*” delivery of hydrogen and the use of Crabtree’s catalyst in a directed hydrogenation for a “*syn*”-addition of hydrogen. Starting from pure enantiomers of limonene [1] these novel synthetic routes provided iridomyrmecins **A**, **A'** and **B**, **B'** in 2–3% yield over 15 steps as well as iridomyrmecins **C**, **C'** and **D**, **D'** in 10–15% yield over 9 steps.

All eight stereoisomers of *trans*-fused iridomyrmecins could be separated by gas chromatography using a capillary column coated with FFAP as an achiral polar stationary phase and a second capillary coated with a 1:1 mixture of OV1701 and heptakis(6-*O*-*tert*-butyldimethylsilyl-2,3-di-*O*-methyl)-β-cyclodextrin as an enantioselective stationary phase. Figure 3 shows the structures and retention times of all eight stereoisomers of *trans*-fused iridomyrmecins on both capillary column systems. Despite the small differences in retention times between **B** and **B'** on the cyclodextrin column, the enantiomers could be well distinguished under the experimental conditions.

### Structure assignment of volatile components **Y** and **Z** in the parasitoid wasp *Alloxysta victrix*

Comparison of mass spectra and GC retention times of synthetic iridomyrmecins with corresponding data of the volatile substances **Y** and **Z** – which are present in pentane extracts of heads of *Alloxysta victrix* – allowed their unambiguous identification as *trans*-fused iridomyrmecins.

**Figure 7:**
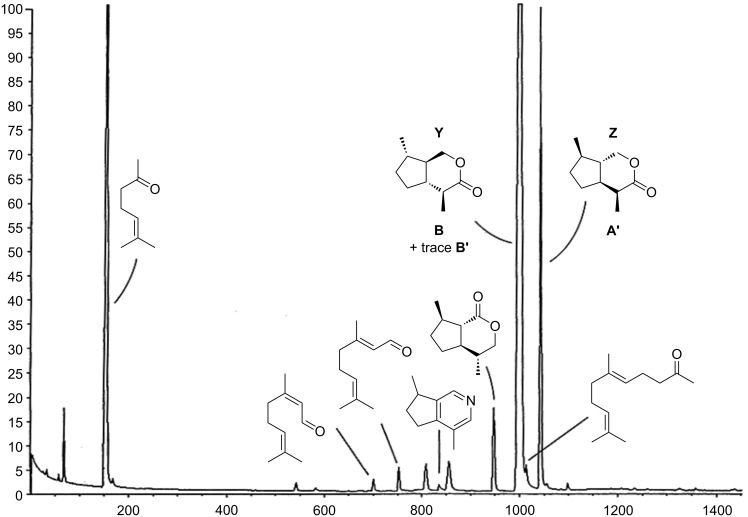
Volatile terpenoids in the cephalic secretion of *Alloxysta victrix*. For the identification of compounds other than **Y** and **Z** see [1].

Coupled GC/MS analysis using FFAP as the stationary phase revealed the natural iridoid lactones **Y** and **Z** to show the same mass spectra and retention times as the two early eluting racemates of the synthetic iridomyrmecins, i.e., **B**/**B'** and **A**/**A'**, respectively (Figure 3). Enantioselective gas chromatography on heptakis(6-*O*-*tert*-butyldimethylsilyl-2,3-di-*O*-methyl)-β-cyclodextrin showed that **A** and **A'** were well separated with an α-value of **A**:**A'** = 1.05 (Figure 3). Consequently, the structure of **Z** could be easily determined to be **A'**, namely (4*S*,4a*S*,7*R*,7a*S*)-iridomyrmecin. Under the same experimental conditions, **B** and **B'** were only poorly resolved, however, heptaiks(2,6-di-*O*-methyl-3-*O*-pentyl)-β-cyclodextrin produced a good α-value of **B'**:**B** = 1.015 [20]. As a result, **Y** was unambiguously identified to be (4*S*,4a*R*,7*S*,7a*R*)-iridomyrmecin. A careful inspection of the analytical data obtained with the cyclodextrin column revealed the presence of small amounts of (4*R*,4a*S*,7*R*,7a*S*)-iridomyrmecin **B'** in the natural extract, showing the ee of natural **B** to be ca. 95–97%. Figure 7 shows a typical gas chromatogram (obtained with FFAP as the stationary phase) of an extract of heads of male *A. victrix*. Identified structures are assigned.

### Structural relations between the *Alloxysta*-compounds and other insect iridoids

**Figure 8:**
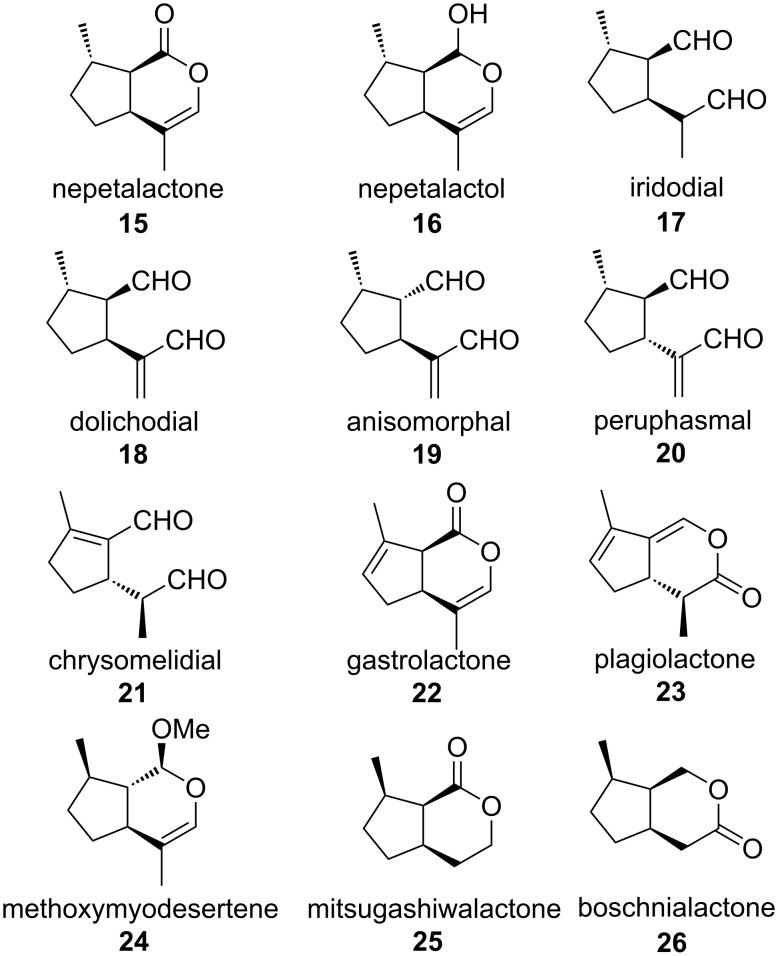
Structures of iridoids from insects and plants. Absolute configurations of **19** and **20** are "educated guesses".

The iridoid lactones which are present in the cephalic secretions of *Alloxysta victrix* show an unusual *trans*-fusion. Among the compounds showing this structure, only the lactone **X** [1] (Figure 1) and a compound with the same relative configuration [21] have been described from insects so far. In contrast to that, the *cis*-fused nepetalactone **15** and diastereomers thereof are typical components of many species of the plant genus *Nepeta* [22–23]. Along with the corresponding hemiacetal **16**, which shows (1*R*)-configuration, **15** is also a most important sex pheromone of aphids [24] (Figure 8). Nevertheless, iridoids are usually associated with defense chemistry. Whilst configurations at the stereogenic centers of **15** and related iridoid lactones in insects appear to be stereotypic, several monocyclic iridoids show further stereochemical variation. Lactol **16** and iridodial (**17**) have first been identified as defense compounds of ants [25]. More recently, **17** – which shows (*R*)-configuration at C1 of the side-chain – was found to be a male-produced aggregation pheromone of lacewings [26–27].

The *cis*-fused iridomyrmecin (**1**) and dolichodial (**18**) are constituents of the anal gland secretion of the Argentine ant *Iridomyrmex humilis* [28]. More recently, (1*S*,2*R*,3*S*)-dolichodial (**18**) has been identified as an electrophysiologically active volatile released from the rosy apple aphid *Dysaphis plantaginea* oviparae and has been discussed apart from **15** and **16** as a possible third component of the aphid sex pheromone [29]. Actually, **18** was first identified in the defensive secretions of dolichoderine ants [25]. The same substance (or its enantiomer) has been found as a defensive compound of *Nematine larvae* [30] and in adults and larvae of the thrips *Calloccithrips fuscipennis* [31]. A stereoisomer of dolichodial – anisomorphal (**19**) – is a component of the defensive secretion of the walking stick *Anisomorpha buprestoides* [32], whilst a third stereoisomer – peruphasmal (**20**) – has been identified in another walking stick, *Peruphasma schultei* [33–34]. Recent investigations show that the qualitative and quantitative composition of cyclopentanoid iridoids in the defensive secretion of *A. buprestoides* may vary with age and population. The secretion may contain all three isomers **18**, **19** and **20** which the insect can produce from glucose [35]. The absolute configurations of **19** and **20** are still unknown. It should be mentioned that along with other iridoids **18** and **19** are also components of the essential oil of some plant species [36]. A related cyclopentene derivative is chrysomelidial (**21**), a relatively widespread defense compound in larvae of phytophagous leaf beetles *Chrysomelidae* [37–38], which has been found in other insects, too. In the defensive secretion of oribatid mites it keeps the depicted (5*S*,8*S*)-configuration [39]. In some species, chrysomelidial is accompanied by the dehydronepetalactone **22** (gastrolactone) [40] or the didehydroiridomyrmecin **23** (plagiolactone) [37]. To the best of our knowledge, the two iridomyrmecins **Y** and **Z** are new natural products, representing the first *trans*-fused iridoid lactones of this type.

Whilst the *trans*-fused nature of the new iridoid lactones is very unusual, their stereochemical pattern (Figure 7) is even more puzzling: The configurational arrangements of the substituents in **X** and **Y** are strictly opposite, whereas the relations between **X** and **Z** are relatively close, showing inversion at C-4 only. Strangely, **Z** is not the expected C-4-epimer of **Y**, but the C-4-epimer of its enantiomer.

Apart from very few exceptions such as methoxymyodesertene (**24**) [41], the 4-*nor*-nepetalactone mitsugashiwalactone (**25**) [42] and its “*nor*-iridomyrmecin-complement” boschnialactone (**26**), [43] which all are plant volatiles, the methyl group in the typical five-membered ring of iridoids keeps its (*S*)-configuration (see also Figure 8), which is just in contrast to **X** and **Z** [44]. However, recently, two stereoisomers of iridomyrmecin showing (7*R*)-configuration have been reported to be components of the defense chemistry of the *Drosophila* parasitoid *Leptopilina heterotoma* [45].

### Remarks on the biosynthesis of iridoids

**Figure 9:**
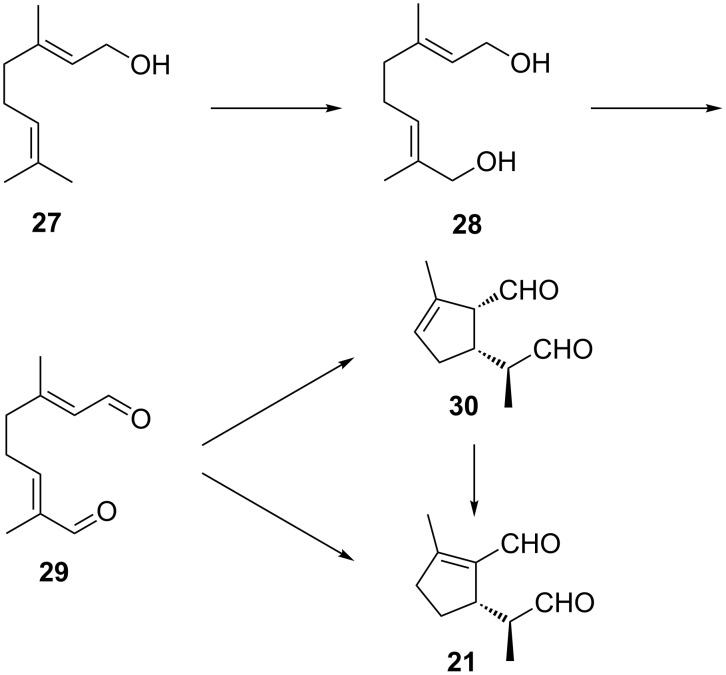
Biosynthetic ways to iridoids from geraniol.

Today it is generally accepted that the biosynthesis of iridoids starts from the acyclic geraniol (**27**). In a series of careful, elegant experiments it was shown that **27** is oxidized to 8-hydroxygeraniol (**28**) which is further transformed to 8-oxogeranial (**29**) [46–48] (Figure 9).

During these investigations the stereochemistry of the subsequent cyclization to iridoids was found to be different in larvae of herbivorous leaf beetles and in carnivorous rove beetles. In *Phaedon cochleariae* (Chrysomelidae), cyclization of **29** directly affords chrysomelidial (**21**). In contrast, in *Phylonthus* sp. (Staphylinidae) the first step is the formation of plagiodial (**30**), which was first identified from larvae of several leaf beetle species [49–50]. Subsequently, **30** may rearrange to the thermodynamically more stable, conjugated **21**. Despite these results, there are still a lot of open questions concerning the biosynthesis of iridoids. Recently, it could be shown that leaf beetles may produce iridoid monoterpenes de novo [33] but they are also able to sequester glycosidically bound terpene precursors from their food plants [51–53] which is highly interesting with regards to the evolution of insect-plant relationships and insect defense chemistry. At present, nothing is known about the formation of “saturated” iridoids such as iridodial (**17**) or the iridoid lactones in *Alloxysta*. The strange stereochemical relations between these compounds may well be the result of different mechanisms in the enzymatic hydrogenation steps.

## Conclusion

The new iridoid lactones have been found in the mandibular gland secretions of several alloxystine wasps [54] and their activity in intraspecific and interspecific communication has been discussed [55]. According to first bioassays, the new iridoids seem to play a multifunctional role in the tritrophic system of the aphid *Sitobion avenae*, its parasite, the wasp *Aphidius uzbekistanicus*, and the hyperparasitoide *Alloxysta victrix* as they obviously sedate *Sitobion* and repell *Aphidius*. However, additional bioassays will be needed to better understand the biological significance of the newly identified iridoid lactones. As our way from limonene to iridoids provides relatively easy access to a large variety of iridoid lactones and monocyclic iridoids, behavior experiments using synthetic compounds may shed some more light on the complex relationships between host aphids, primary parasitoids such as *Aphidius* spp., and aphid hyperparasitoids such as *Alloxysta* spp. [56].

## Supporting Information

File 1 Experimental details and characterization data for synthesized compounds.
