# Supplementary material for: Stereoselective synthesis of trans-fused iridoid lactones and their identification in the parasitoid wasp Alloxysta victrix, Part II: Iridomyrmecins
Source: Beilstein J Org Chem. 2012 Aug 8;8:1256–64. doi: 10.3762/bjoc.8.141 (PMC3458746; doi:10.3762/bjoc.8.141)
Supplement: File 1 — Experimental details and characterization data for synthesized compounds. [file Beilstein_J_Org_Chem-08-1256-s001.pdf]

Supporting Information

for

Stereoselective synthesis of *trans*-fused iridoid lactones and their identification in the parasitoid wasp *Alloxysta victrix*, Part II: Iridomyrmecins

Robert Hilgraf, Nicole Zimmermann, Lutz Lehmann, Armin Tröger and Wittko Francke*

Address: Department of Chemistry - Organic Chemistry, University of Hamburg, Martin-Luther-King-Platz 6, D-20146 Hamburg, Germany

Email: Wittko Francke - francke@chemie.uni-hamburg.de

* Corresponding author

Experimental details and characterization data for synthesized compounds

General remarks:

^{1}H and ^{13}C NMR spectra were recorded with a Bruker DRX 500 (^{1}H: 500 MHz, ^{13}C: 126 MHz) or with a Bruker AMX 400 (^{1}H: 400 MHz, ^{13}C: 101 MHz) spectrometer. Chemical shifts were referenced to the corresponding residual solvent signal, i.e., δ_H = 7.26 and δ_C = 77.0 ppm for $CDCl_3$. Coupled gas chromatography/mass spectrometry (GC/MS) analyses at low resolution (electron impact (EI) at 70 eV) using a Fisons GC8008/MD800 equipped with an Optima 5MS column (30 m, 0.25 m id fused silica capillary, Macherey-Nagel, Düren, Germany) 3 min at 50 °C, then programmed to 220 °C at a rate of 5 °C/min using helium as the carrier gas. High resolution GC/MS analyses (GC/HRMS–EI, RP:5000) and GC/CIMS (RP:600) were carried out by using a double focusing mass spectrometer VG 70/250 SE (Vacuum Generators, Manchester, UK) linked to a gas chromatograph HP 5890 (Hewlett Packard, Palo Alto, CA, USA). Separation conditions were the same as those used for low resolution GC/MS. Standard gas chromatography was carried out by using a Satochrom (Fisons instruments) equipped with fused silica capillaries. Hydrogen served as the carrier gas. The following columns were employed: a) FFAP (50 m, 0.25 mm id, Macherey-Nagel, Düren, Germany) 3 min at 50 °C, then programmed to 220 °C at a rate of 5 °C/min; b) 1:1 mixture of OV1701 and heptakis-(6-*O*-*tert*-butyldimethylsilyl-2,3-di-*O*-methyl)-β-cyclodextrin (25 m, 0.25 mm id, tailor-made at our lab) 5 min at 60 °C , then programmed to 160 °C at a rate of 3 °C/min. Flash chromatography was carried out using Merck silica gel 60 (240–400 mesh). All experiments were carried out in oven-dried glassware under a dry argon atmosphere. Standard vacuum techniques were used for the handling of air-sensitive materials. Solvents were dried and kept under N_2 and freshly distilled before use. Reagents were used as commercially available.

(1*R*,2*R*,5*S*)-1-Hydroxymethyl-2-(2-*tert*-butyldimethylsilyloxy-(1*R*)-methylethyl)-5-methylcyclopentane (9): To a stirred solution of the aldehyde **8** [1] (5.7 g, 20.0 mmol) in 180 mL of methanol was added a solution of sodium borohydride (3.82 g, 101 mmol) in water (37 mL), and stirring was continued for 90 min at rt. The reaction mixture was quenched by careful addition of 0.5 M hydrochloric acid, and the aqueous phase was extracted with diethyl ether (4 x 300 mL). The combined organic solutions were dried over magnesium sulfate and concentrated in vacuo to give alcohol **9** (4.53 g, 79%) as a pale yellow oil. ^{1}H NMR (400 MHz, $CDCl_3$): δ = −0.01 (s, 6H, $Si(CH_3)_2$), 0.76 (d, $^3J_{H,H}$ = 7.1 Hz, 3H, 1'-CH_3), 0.84 (s, 9H, $C(CH_3)_3$), 0.94 (d, $^3J_{H,H}$ = 6.6 Hz, 3H, 5-CH_3), 1.17 (m, 2H), 1.43 (m, 2H), 1.64 (m, 3H), 1.77 (m, 1H), 1.90 (m, 1H), 3.43 (m, 3H, 2'-H_a and 1-CH_2), 3.53 (dd, $^3J_{H,H}$ = 5.1,10.7 Hz, 1H, 2'-H_b); ^{13}C NMR (126 MHz, $CDCl_3$): δ = −4.9, 14.1, 20.1, 26.3, 29.6, 32.0, 34.4, 38.2, 39.6, 44.2, 50.7, 67.1, 67.2; MS *m*/*z* (%): 286 (0) $[M]^+$, 137 (36), 105 (35), 95 (73), 81 (83), 75 (100), 73 (33), 57 (22), 55 (25), 41 (32); Anal. calcd for $C_{16}H_{34}O_2Si$: C, 67.07; H, 11.96; found: C, 67.1; H, 12.00.

(1*S*,2*S*,5*R*)-1-Hydroxymethyl-2-(2-*tert*-butyldimethylsilyloxy-(1*S*)-methylethyl)-5-methylcyclopentane (9'): Applying the same procedure to **8'** [1] (7.8 g, 27.4 mmol) yielded 6.82 g (87%) of **9'** as an oil. The NMR and mass spectra were identical to those of **9**.

(1*R*,2*R*,5*S*)-1-Benzyloxymethyl-2-(2-*tert*-butyldimethylsilyloxy-(1*R*)-methylethyl)-5-methylcyclopentane (10): Alcohol **9** (4.4 g, 15.4 mmol) was added dropwise to a stirred suspension of sodium hydride (60%, 464 mg, 19.3 mmol) in 9 mL of tetrahydrofuran. After stirring for 1 h at rt, benzyl bromide (1.76 g, 16.1 mmol) was added, and the suspension was heated under reflux for 1 h. After cooling to rt the

reaction was quenched by careful addition of water. The aqueous phase was extracted with diethyl ether (5 x 50 mL). The combined organic phases were dried over magnesium sulfate and concentrated to give the benzyl ether **10** (5.55 g, 96%) as a pale yellow oil. ^{1}H NMR (400 MHz, $CDCl_3$): δ = −0.01 (s, 6H, $Si(CH_3)_2$), 0.86 (s, 9H, $C(CH_3)_3$), 0.88 (d, $^{3}J_{H,H}$ = 7.1 Hz, 3H, 3-H), 0.98 (d, $^{3}J_{H,H}$ = 6.6 Hz, 3H, 3'-CH_3), 1.50 (m, 8H), 3.44 (m, 4H, 2'-CH_2 and 1-H), 4.48 (d, $^{3}J_{H,H}$ = 3.6 Hz, 2H, CH_2Ph), 7.31 (m, 5H, H_{Ar}); ^{13}C NMR (101 MHz, $CDCl_3$): δ = −5.0, 16.2, 21.0, 26.4, 28.8, 32.0, 34.2, 38.6, 40.4, 45.2, 50.2, 67.1, 73.4, 74.5, 127.8, 128.7; MS *m*/*z* (%): 376 (0) $[M]^+$, 92 (12), 91 (100), 81 (13), 75 (15), 73 (14); Anal. calcd for $C_{23}H_{40}O_2Si$: C, 73,34; H, 10.70; found: C, 73.3; H, 10.73%.

(1*S*,2*S*,5*R*)-1-Benzyloxymethyl-2-(2-*tert*-butyldimethylsilyloxy-(1*S*)-methylethyl)-5-methylcyclopentane (10'): Applying the same procedure to **9'** (5.82 g, 20.3 mmol) yielded 7.28 g (95%) of **10'** as an oil. The NMR and mass spectra were identical to those of **10**.

(1*R*,2*S*,5*S*)-1-Benzyloxymethyl-2-(2-hydroxy-(1*R*)-methylethyl)-5-methylcyclopentane (11): Benzyl ether **10** (6.68 g, 17.8 mmol) was dissolved in 32 mL of acetonitrile, and 40% aqueous hydrofluoric acid (1.8 mL) was added at rt. After stirring for 30 min at rt, the reaction was quenched by the addition of saturated aqueous sodium hydrogen carbonate (50 mL). The aqueous phase was extracted with diethyl ether (3 x 60 mL), and the combined organic phases were washed with brine (30 mL) and dried over magnesium sulfate. The solvent was removed in vacuo, and the residue was purified by chromatography over silica (hexane/ethyl acetate 8:1, then 3:1) to give benzyl ether **11** (3.30 g, 71%) as a pale yellow oil. ^{1}H NMR (400 MHz, $CDCl_3$): δ = 0.84 (d, $^{3}J_{H,H}$ = 7.1 Hz, 3H, 3-H), 0.99 (d, $^{3}J_{H,H}$ = 6.6 Hz, 3H,

3'-CH_3), 1.21 (m, 1H), 1.48 (m, 1H), 1.65 (m, 4H), 1.80 (m, 1H), 1.97 (br s, 1H, OH), 3.31 (dd, $^3J_{H,H}$ = 8.0, 11.2 Hz, 1H, 1-H_a), 3.40 (dd, $^3J_{H,H}$ = 5.6, 11.2 Hz, 1H, 1-H_b), 3.50 (m, 2H, 2'-CH_2), 4.53 (d, $^3J_{H,H}$ = 3.1 Hz, 2H, CH_2Ph), 7.33 (m, 5H, H_{Ar}); ^{13}C NMR (101 MHz, $CDCl_3$): δ = 13.7, 19.9, 29.4, 34.0, 38.4, 39.5, 44.3, 47.3, 66.4, 73.4, 74.9, 127.8, 128.5; MS *m*/*z* (%): 262 (1) $[M]^+$, 123 (24), 107 (50), 95 (32), 92 (21), 91 (100), 81 (39), 55 (34), 41 (21); Anal. calcd for $C_{17}H_{26}O_2$: C, 77.82; H, 9.99; found: C, 77.7; H, 9.95%.

(1*S*,2*R*,5*R*)-1-Benzyloxymethyl-2-(2-hydroxy-(1*S*)-methylethyl)-5-methylcyclopentane (11'): Applying the same procedure to **10'** (7.23 g, 19.2 mmol) yielded 3.16 g (63%) of **11'** as an oil. The NMR and mass spectra were identical to those of **11**.

(1*R*,2*R*,5*S*)-1-Benzyloxymethyl-2-[(1*R*)-carboxyethyl]-5-methylcyclopentane (12): Benzyl ether **11** (3.3 g, 12.6 mmol) was dissolved in 30 mL of acetone, and 5 mL of 8 N Jones reagent was added dropwise at rt. Stirring was continued for 30 min at rt, and after removal of the solvent in vacuo, the residue was dissolved in diethyl ether (45 mL), and water (55 mL) was added. The phases were separated, and the aqueous phase was extracted with diethyl ether (4 x 50 mL). The combined organic phases were dried over magnesium sulfate and concentrated in vacuo to give benzyl ether **12** (3.44 g, 99%) as a pale yellow oil. ^{1}H NMR (400 MHz, $CDCl_3$): δ = 0.99 (d, $^3J_{H,H}$ = 6.6 Hz, 3H, 3'-CH_3), 1.19 (m, 5H), 1.51 (m, 2H), 1.71 (m, 3H), 1.92 (m, 1H), 3.46 (m, 3H, 2'-CH_2 and 2-H), 4.54 (s, 2H, CH_2Ph), 7.33 (m, 5H, H_{Ar}); ^{13}C NMR (101 MHz, $CDCl_3$): δ = 15.8, 19.9, 27.4, 32.6, 37.5, 42.7, 47.2, 50.2, 65.9, 73.5, 127.6, 128.5; MS *m*/*z* (%): 276 (1) $[M]^+$, 125 (16), 122 (10), 95 (14), 92 (12), 91

(100), 82 (14), 79 (10), 71 (11), 70 (12), 67 (15), 65 (12), 55 (10), 43 (66); Anal. calcd for $C_{17}H_{24}O_3$: C, 73,88; H, 8.75; found: C, 73.8; H, 8.71%.

(1*S*,2*S*,5*R*)-1-Benzyloxymethyl-2-[(1*S*)-carboxyethyl]-5-methylcyclopentane (12'): Applying the same procedure to **11'** (3.16 g, 12.0 mmol) yielded 3.30 g (99%) of **12'** as an oil. The NMR and mass spectra were identical to those of **12**.

(1*R*,2*R*,5*S*)-1-Hydroxymethyl-2-[(1*R*)-carboxyethyl]-5-methylcyclopentane (5): The benzyl ether **12** (3.39 g, 12.3 mmol) was dissolved in THF (40 mL), and 10% Pd/C (400 mg) was added. The reaction mixture was hydrogenated at a pressure of 40 bar for 30 min. The suspension was filtered over Celite and washed with diethyl ether 1 L). The solvent was concentrated in vacuo to give the hydroxy acid **5** as a pale yellow oil (quantitative yield), which was used without further purification in the next step; MS *m*/*z* (%): 186 (0) $[M]^+$, 95 (100), 94 (23), 81 (68), 79 (26), 74 (67), 67 (60), 55 (49), 53 (29), 45 (26), 41 (69), 39 (40); Anal. calcd for $C_{10}H_{18}O_3$: C, 64.49; H, 9.74; found: C, 64.6; H, 9.77%.

(1*S*,2*S*,5*R*)-1-Hydroxymethyl-2-[(1*S*)-carboxyethyl]-5-methylcyclopentane (5'): Applying the same procedure to **12'** (3.30 g, 11.9 mmol) afforded **5'** as an oil. The mass spectrum was identical to that of **8**.

(4*R*,4a*R*,7*S*,7a*R*)-4,7-Dimethylhexahydrocyclopenta[*c*]pyran-3(1*H*)-one (iridomyrmecin A): To a solution of the crude hydroxy acid **5** (1.15 g, 6.18 mmol) in 400 mL of dichloromethane was added dicyclohexylcarbodiimide (1.39 g, 6.74 mmol) and dimethylaminopyridine (97 mg, 0.79 mmol). The reaction mixture was stirred at rt for 75 min. The solvent was removed in vacuo, and the residue was suspended in

hexane (130 mL). The resulting precipitate was filtered off and washed with hexane (3 x 100 mL). The filtrate was concentrated in vacuo, and the residue was purified by chromatography over silica (hexane/ethyl acetate, 5:1) to give iridomyrmecin **A** (690 mg, 66%) as a pale yellow oil. ^{1}H NMR (500 MHz, $CDCl_3$): δ = 1.05 (d, $^{3}J_{H,H}$ = 6.6 Hz, 3H, 7-CH_3), 1.24 (d, $^{3}J_{H,H}$ = 7.5 Hz, 3H, 4-CH_3), 1.40 (m, 1H, 6-H_a), 1.52 (m, 1H, 5-H_b), 1.59 (br dddd, 1H, 7a-H), 1.66 (m, 2H, 5-H_a and 7-H), 2.06 (m, 2H, 6-H_b and 4a-H), 2.92 (dq, $^{3}J_{H,H}$ = 6.0, 7.6 Hz, 1H, 4-H), 4.02 (dd, $^{3}J_{H,H}$ = 10.5, 10.7 Hz, 1H, 1-H_b), 4.54 (dd, $^{3}J_{H,H}$ = 5.1, 10.5 Hz, 1H, 1-H_a); ^{13}C NMR(126 MHz, $CDCl_3$): δ = 13.1, 19.2, 24.5, 33.2, 36.4, 38.9, 42.3, 44.7, 74.7, 175.0; MS *m*/*z* (%): 168 (0) $[M]^+$, 110 (18), 95 (44), 82 (30), 81 (95), 79 (19), 77 (10), 69 (20), 68 (50), 67 (100), 55 (33), 54 (11), 53 (27), 41 (74), 40 (11), 39 (51); Anal. calcd for $C_{10}H_{16}O_2$: C, 71,39; H, 9.59; found: C, 71.3; H, 9.63%.

(4*S*,4a*S*,7*R*,7a*S*)-4,7-Dimethylhexahydrocyclopenta[*c*]pyran-3(1*H*)-one (iridomyrmecin A'): Applying the same method to **5'** (1.20 g, 6.44 mmol) yielded 678 mg (63%) of iridomyrmecin **A'** as an oil. The NMR and mass spectra were identical to those of iridomyrmecin **A**.

(4*S*,4a*R*,7*S*,7a*R*)-4,7-Dimethylhexahydrocyclopenta[*c*]pyran-3(1*H*)-one (iridomyrmecin B): A mixture of the crude hydroxy acid **5** (1.15 g, 6.18 mmol) and *p*-toluenesulfonic acid (230 mg, 1.32 mmol) in 103 mL of benzene was heated under reflux overnight. After cooling to rt, diethyl ether (50 mL) and water (500 mL) were added, and the phases were separated. The aqueous phase was extracted with diethyl ether (3 x 75 mL). The combined organic phases were washed with 30 mL of brine, dried over magnesium sulfate, and concentrated in vacuo. The residue was purified by chromatography over silica (hexane/ethyl acetate, 5:1) to give

iridomyrmecin **B** (583 mg, 56%) as a pale yellow oil. ^{1}H NMR (500 MHz, $CDCl_3$): δ = 1.04 (d, $^3J_{H,H}$ = 6.6 Hz, 3H, 7-CH_3), 1.28 (d, $^3J_{H,H}$ = 6.9 Hz, 3H, 4-CH_3), 1.29 (m, 1H, 5-H_a), 1.42 (m, 1H, 6-H_a), 1.49 (br dddd, 1H, 7a-H), 1.64 (br dddd, $^3J_{H,H}$ = 11.7 Hz, 1H, 4a-H), 1.73 (m, 1H, 7-H), 1.96 (m, 1H, 5-H_b), 2.08 (m, 1H, 6-H_a), 2.27 (dq, $^3J_{H,H}$ = 6.9, 11.7 Hz, 1H, 4-H), 4.06 (dd, $^3J_{H,H}$ = 10.4, 11.0 Hz, 1H, 1-H_b), 4.54 (dd, $^3J_{H,H}$ = 5.1, 10.4 Hz, 1H, 1-H_a); ^{13}C NMR(126 MHz, $CDCl_3$): δ = 15.4, 19.0, 29.0, 33.1, 36.7, 44.2, 48.4, 48.8, 74.3, 174.0; MS *m/z* (%): 168 (1) $[M]^+$, 109 (14), 95 (33), 82 (23), 81 (86), 79 (15), 69 (22), 68 (52), 67 (100), 55 (27), 53 (20), 41 (68), 40 (10), 39 (42); Anal. calcd for $C_{10}H_{16}O_2$: C, 71.39; H, 9.59; found: C, 71.3; H, 9.55%.

(4*R*,4a*S*,7*R*,7a*S*)-4,7-Dimethylhexahydrocyclopenta[*c*]pyran-3(1*H*)-one (iridomyrmecin B’): Applying the same method to crude **5’** (1.20 g, 6.44 mmol) yielded 493 mg (45%) of iridomyrmecin **B’** as an oil. The NMR and mass spectra were identical to those of iridomyrmecin **B**.

(1*R*,2*R*,5*R*)-1-Acetoxy-2-[(1*R*)-carboxyethyl]-5-methylcyclopentane (14): To a solution of the acetate **13** [1] (5.0 g, 23.3 mmol) in acetone (50 mL) was slowly added 10 mL of 8 N Jones reagent at 0 °C. The reaction mixture was stirred for 1 h at rt. The solvent was removed in vacuo, the residue was dissolved in diethyl ether (80 mL) and washed with water (100 mL). The aqueous phase was extracted with diethyl ether (4 x 90 mL). The combined organic phases were washed with 150 mL of brine, dried over magnesium sulfate, and concentrated in vacuo to give the acid **14** (4.59 g, 86%) as a pale yellow oil. ^{1}H NMR (400 MHz, $CDCl_3$): δ = 0.94 (d, $^3J_{H,H}$ = 6.6 Hz, 3H, 3’-CH_3), 1.22 (d, $^3J_{H,H}$ = 7.1 Hz, 3H, 3-H), 1.30 (m, 2H), 1.40 (m, 2H), 1.74 (m, 1H), 1.92 (m, 1H), 2.10 (m, 1H), 2.48 (m, 1H, 1’-H), 2.50 (s, 3H, Ac-CH_3), 3.49 (m, $^3J_{H,H}$ = 7.1 Hz, 1H, 2-H), 3.98 (dd, $^3J_{H,H}$ = 6.4, 10.7 Hz, 1H, 2’-CH_a), 4.09

(dd, $^3J_{H,H}$ = 6.6, 10.7 Hz, 1H, 2'-CH_b); ^{13}C NMR (101 MHz, $CDCl_3$): δ = 15.0, 15.7, 21.0, 28.9, 33.4, 36.3, 43.5, 43.7, 44.5, 65.5, 171.3, 181.4; MS *m*/*z* (%): 228 (0) $[M]^+$, 95 (100), 94 (20), 81 (34), 79 (12), 67(16), 55 (13), 43 (70), 41 (20); Anal. calcd for $C_{12}H_{20}O_4$: C, 63.17; H, 8.83; found: C, 63.1; H, 8.79%.

(1*S*,2*S*,5*S*)-1-Acetoxy-2-[(1*S*)-carboxyethyl]-5-methylcyclopentane (14'): Applying the same method to **13'** [1] (5.0 g, 23.3 mmol) yielded 4.80 g (90%) of **14'** as an oil. The NMR and mass spectra were identical to those of **14**.

(1*R*,2*R*,5*R*)-1-Hydroxymethyl-2-[(1*R*)-carboxyethyl]-5-methylcyclopentane (6): The acetate **14** (4.4 g, 19.1 mmol) was dissolved in 20 mL of methanol and added dropwise to 120 mL of a 2 M solution of potassium hydroxide in methanol at 0 °C. After stirring for 2 h at rt the reaction mixture was poured into ice water (80 mL) and extracted twice with diethyl ether (80 mL). The aqueous phase was acidified with 5 M hydrochloric acid and extracted with diethyl ether (4 x 80 mL). The combined organic layers were washed with brine (80 mL), dried over magnesium sulfate, and concentrated in vacuo to give hydroxy acid **6** (3.47 g, 97%) as a pale yellow oil which was used without further purification in the next step; MS *m*/*z* (%): 186 (0) $[M]^+$, 95 (82), 94 (23), 81 (100), 79 (23), 74 (56), 68 (20), 67 (80), 55 (51), 53 (31), 41 (59), 39 (52); Anal. calcd for $C_{10}H_{18}O_3$: C, 64.49; H, 9.74; found: C, 64.4; H, 9,77%.

(1*S*,2*S*,5*S*)-1-Hydroxymethyl-2-[(1*S*)-carboxyethyl]-5-methylcyclopentane (6'): Applying the same procedure to **14'** (4.70 g, 20.7 mmol) yielded 3.52 g (92%) of crude **6'** as an oil. The mass spectrum was identical to that of **6**.

(4*R*,4a*R*,7*R*,7a*R*)-4,7-Dimethylhexahydrocyclopenta[*c*]pyran-3(1*H*)-one (iridomyrmecin C): To a solution of the crude hydroxy acid **6** (3.07 g, 16.5 mmol) in 1250 mL of dichloromethane was added dicyclohexylcarbodiimide (3.72 g, 18.0 mmol) and dimethylaminopyridine (250 mg, 2.1 mmol). The reaction mixture was stirred for 75 min at rt. The solvent was removed in vacuo, and the residue was suspended in 350 mL of hexane. The precipitate was filtered off and washed with hexane (4 x 250 mL). The solvent was removed in vacuo, and the residue was purified by chromatography over silica (hexane/diethyl acetate, 3:1) to give iridomyrmecin **C** (1.14 g, 41%) as a pale yellow oil. ^{1}H NMR (500 MHz, $CDCl_3$): δ = 0.87 (d, $^{3}J_{H,H}$ = 7.3 Hz, 3H, 7-CH_3), 1.23 (d, $^{3}J_{H,H}$ = 7.6 Hz, 3H, 4-CH_3), 1.34 (m, 1H, 6-H_b), 1.43 (m, 1H, 5-H_b), 1.72 (m, 1H, 5-H_a), 2.05 (br dddd, 1H, 7a-H), 2.12 (m, 1H, 6-H_a), 2.19 (br dddd, $^{3}J_{H,H}$ = 6.3 Hz, 1H, 4a-H), 2.35 (dddq, $^{3}J_{H,H}$ = 7.6 Hz, 1H, 7-H), 2.99 (dq, $^{3}J_{H,H}$ = 7.6 Hz, 1H, 4-H), 4.20 (dd, $^{3}J_{H,H}$ = 10.5, 11.8 Hz, 1H, 1-H_b), 4.48 (dd, $^{3}J_{H,H}$ = 5.1, 10.5 Hz, 1H, 1-H_a); ^{13}C NMR(126 MHz, $CDCl_3$): δ = 13.2, 17.1, 26.1, 31.8, 34.1, 37.3, 38.9, 41.0, 72.5, 175.1; MS *m*/*z* (%): 168 (1) $[M]^+$, 95 (43), 82 (29), 81 (98), 69 (10), 68 (58), 67 (*100*), 55 (32), 53 (17), 41 (66), 39 (47); Anal. calcd for $C_{10}H_{16}O_2$: C, 71.39; H, 9.59; found: C, 71.3; H, 9.54%.

(4*S*,4a*S*,7*S*,7a*S*)-4,7-Dimethylhexahydrocyclopenta[*c*]pyran-3(1*H*)-one (iridomyrmecin C'): Applying the same method to crude **6'** (3.90 g, 15.6 mmol) yielded 1.39 g (39%) of iridomyrmecin **C'** as an oil. The NMR and mass spectra were identical to those of iridomyrmecin **C**.

(4*S*,4a*R*,7*R*,7a*R*)-4,7-Dimethylhexahydrocyclopenta[*c*]pyran-3(1*H*)-one (iridomyrmecin D): A solution of the hydroxy acid **6** (505 mg, 2.71 mmol) and *p*-toluenesulfonic acid (100 mg, 0.53 mmol) in benzene (45 mL) was heated under

reflux overnight. After cooling to rt, diethyl ether (100 mL) and water (100 mL) were added, and the phases were separated. The aqueous phase was extracted with diethyl ether (4 x 75 mL). The combined organic phases were washed with 30 mL of brine, dried over magnesium sulfate and concentrated in vacuo. The residue was purified by chromatography over silica (hexane/ethyl acetate, 5:1) to give iridomyrmecin **D** (270 mg, 59%) as a pale yellow oil. ^{1}H NMR (500 MHz, $CDCl_3$): δ = 0.85 (d, $^{3}J_{H,H}$ = 7.6 Hz, 3H, 7-CH_3), 1.18 (d, 1H, 5-H_a), 1.31 (d, $^{3}J_{H,H}$ = 7.1 Hz, 3H, 4-CH_3), 1.38 (m, 1H, 6-H_a), 1.79 (br dddd, 1H, 4a-H), 1.91 (br dddd, 1H, 7a-H), 2.04 (m, 1H, 5-H_b), 2.15 (m, 1H, 6-H_a), 2.25 (dq, $^{3}J_{H,H}$ = 7.1, 11.2 Hz, 1H, 4-H), 2.37 (dddq, $^{3}J_{H,H}$ = 7.6 Hz, 1H, 7-H), 4.23 (dd, $^{3}J_{H,H}$ = 10.7, 12.2 Hz, 1H, 1-H_b), 4.49 (dd, $^{3}J_{H,H}$ = 5.6, 10.7 Hz, 1H, 1-H_a); ^{13}C NMR(126 MHz, $CDCl_3$): δ = 15.7, 17.2, 30.3, 32.6, 33.7, 44.2, 44.5, 44.6, 72.2, 174.0; MS *m*/*z* (%): 168 (1) $[M]^+$, 109 (15), 95 (33), 82 (25), 81 (100), 79 (18), 77 (10), 69 (23), 68 (48), 67 (94), 55 (32), 54 (11), 53 (28), 41 (68), 40 (11), 39 (48); Anal. calcd for $C_{10}H_{16}O_2$: C, 71.39; H, 9.59; found: C, 71.3; H, 9.62%.

(4*R*,4a*S*,7*S*,7a*S*)-4,7-Dimethylhexahydrocyclopenta[*c*]pyran-3(1*H*)-one (iridomyrmecin D'): Applying the same procedure to crude **6'** (361 mg, 1.80 mmol) yielded 143 mg (47%) of iridomyrmecin **D'** as an oil. The NMR and mass spectra were identical to those of iridomyrmecin **D**.

References

1. Zimmermann, N.; Hilgraf, R.; Lehmann, L.; Ibarra, D.; Francke, W. *Beilstein J. Org. Chem.* **2012,** *8,* 1246–1255.
